# *In situ* propagation-based lung computed tomography for large animal models

**DOI:** 10.1107/S160057752500832X

**Published:** 2025-10-27

**Authors:** Lorenzo D’Amico, Lucy Costello, Yakov Nesterets, Martin Donnelley, Timur Gureyev, Anton Maksimenko, Cathy Beck, Jannis Ahlers, Ronan Smith, Ying Ying How, David Parsons, Chris Hall, Daniel Hausermann, Matthew Cameron, Mitzi Klein, Marcus Kitchen, Giuliana Tromba, Christian Dullin, Kaye Morgan

**Affiliations:** a School of Physics and Astronomy Monash University Clayton Victoria Australia; b Elettra-Sincrotrone Trieste SCpA Trieste Italy; c Commonwealth Scientific and Industrial Research Organisation (CSIRO) Clayton Victoria Australia; d Adelaide Medical School and Robinson Research Institute University of Adelaide Adelaide South Australia Australia; e Respiratory Medicine Women’s and Children’s Hospital Adelaide Adelaide South Australia Australia; f School of Physics The University of Melbourne Parkville Victoria Australia; g Australian Synchrotron, Australian National Science and Technology Organisation, Clayton, Australia; h Melbourne Veterinary School Faculty of Science University of Melbourne Melbourne Victoria Australia; i Diagnostic and Interventional Radiology, University Medicine Goettingen, Goettingen, Germany; j Diagnostic and Interventional Radiology University Hospital Heidelberg Heidelberg Germany; k Translational Molecular Imaging Max Planck Institute for Multidisciplinary Sciences Goettingen Germany; Tohoku University, Japan

**Keywords:** *in situ*, lung imaging, propagation-based phase contrast, synchrotron, computed tomography

## Abstract

The lung is a complex organ with a hierarchical structure, containing four times more air than tissue. It is in constant contact with environmental factors such as pollution and pathogens, leading to pathological alterations at various hierarchical levels. Because of its intricate structure and continuous movement, lung imaging presents significant challenges for most existing techniques. Recent advancements in phase-contrast computed tomography and photon-counting detectors have greatly enhanced lung imaging capabilities. Specifically, propagation-based imaging (PBI), a phase-contrast method that does not require optical elements, has proven particularly effective at low X-ray dose rates due to the strong phase shifts between lung tissue and aerated regions. This study introduces an *in situ* imaging approach for large-scale lungs using PBI at the Imaging and Medical Beamline (IMBL) of the Australian Synchrotron. We investigated optimal conditions for PBI, including energy and propagation distance settings, and found that an X-ray beam energy of 70 keV combined with a 7 m propagation distance yields the highest image quality in terms of contrast-to-noise ratio while also delivering the lowest radiation dose. Furthermore, Monte Carlo simulations were performed on the reconstructed volume to calculate absorbed radiation doses in tissues. These findings provide valuable insights for designing future experiments aimed at minimizing radiation exposure and potentially enable *in vivo* applications in larger animals or even humans.

## Abstract 2

A method for *in situ* imaging of human-sized animal lungs using the Imaging and Medical Beamline (IMBL) at the Australian Synchrotron is presented.

## 1. Introduction

The lung is one of the most complex organs in the body. A human lung with a volume of approximately 1/2 L of tissue can enclose 4 L of air (Weibel, 2009 ▸), and this air makes visualization of the lung challenging for most medical imaging modalities. The lung architecture comprises millions of air-filled spaces surrounded by soft tissue (alveoli), providing a high surface area for gas exchange, but presenting challenges in terms of spatially resolving these structures in images. Over the last decade, advances in X-ray phase-contrast computed tomography (PC CT) (Snigirev *et al.*, 1995 ▸; Momose *et al.*, 1996 ▸; Bonse & Busch, 1996 ▸; Baruchel *et al.*, 2000 ▸) have taken advantage of the strong refractive index gradients created by the numerous soft tissue/air interfaces within the lung to improve lung imaging capabilities (Yagi *et al.*, 1999 ▸). Phase contrast can provide superior image quality, increasing the contrast-to-noise ratio (CNR) by up to two orders of magnitude compared with classical attenuation-based CT (Nesterets & Gureyev, 2014 ▸; Kitchen *et al.*, 2017 ▸; Albers *et al.*, 2023 ▸). This boost in CNR also enables a reduction in radiation dose, making the technique more dose-efficient (Pollock *et al.*, 2024 ▸). The lung is particularly well suited to PC CT, since the difference in the refractive index between the lung tissue and the aerated areas causes strong phase shifts. Several methods have been developed to exploit the information within those phase shifts, including the simplest approach known as propagation-based imaging (PBI), which does not require any optical elements (*e.g.* crystals or gratings). However, to use PBI some requirements need to be fulfilled. These include sufficient spatial coherence, a large propagation distance between the sample and detector (compared with classic attenuation-based CT), and the use of high-resolution detectors to resolve the interference fringes generated by the phase shifts. The advent of synchrotron facilities, which can generate X-rays that fulfill the aforementioned conditions, has paved the way for PBI-based lung imaging.

So far, PBI has been successfully used to perform *in vivo* lung imaging in small animals such as mice (Dullin *et al.*, 2024*a* ▸), rats (Lovric *et al.*, 2017 ▸) and rabbits (Kitchen *et al.*, 2005 ▸). However, these models vary significantly in size and structure from the lungs of larger animals and humans (West, 2012 ▸).

To develop further lung imaging strategies and to evaluate their performance and benefits for large lungs, chest phantoms are often used before imaging live animals or humans. Those phantoms can be entirely artificial, such as the LungMan (Kyoto Kagaku) (Rodríguez Pérez *et al.*, 2018 ▸; Costello *et al.*, 2025 ▸; Ahlers *et al.*, 2025 ▸), or hybrid models consisting of a thorax-shaped enclosure that can accommodate freshly excised large animal lungs (Dullin *et al.*, 2024*b* ▸). LungMan has been designed to resemble the radiological appearance of a human chest when using conventional X-ray and CT techniques. Despite significant advances in material engineering to mimic the refractive index of human ribs and bones, these phantoms, while useful, are still far from replicating the natural conditions in which the lungs are typically found. Moreover, PBI and high-resolution imaging are highly sensitive to material properties, making real tissue more suitable than phantoms for parameter optimization. The use of hybrid phantoms also comes with several drawbacks, including the need for large animal lungs (*e.g.* pigs or sheep) to be extracted and carefully handled during setup to reduce the risk of rupture. Both types of phantoms are also expensive, limiting their availability to many researchers or institutions. Working with them also requires specific expertise, adding additional challenges to their use.

Under physiological conditions, the lung remains inflated by a complex interplay between its internal fiber structure and the negative pressure generated within the chest cavity by the diaphragm and thoracic muscles (Weibel, 2009 ▸). Therefore, it is difficult to study the lungs outside the chest, as they would collapse and deform.

Another approach for lung imaging is the use of explanted lungs without a phantom, which requires the use of a tissue preservation method. However, it is nearly impossible to fix the lung structure while maintaining physiological shape. In addition, the process is time-consuming, requiring several hours of preparation, and even minor errors during this period can compromise the results. The fixation, typically performed using formalin, can also lead to tissue shrinkage, which adds complexity to data interpretation. Knudsen *et al.* (2023 ▸) demonstrated that variations in lung inflation pressure can lead to unfolding, stretching and structural changes in the tissue during the fixation process. Consequently, selecting the appropriate pressure adds another layer of complexity to the tissue stabilization (Knudsen *et al.*, 2023 ▸). After fixation, the lungs must be stabilized for imaging, typically by submerging them in ethanol, which again is challenging and bears the risk of generating air bubbles that compromise image quality in PBI. However, this approach has been successfully applied for hierarchical PBI imaging at the European Synchrotron Radiation Facility (ESRF) on a whole lung (Walsh *et al.*, 2021 ▸). The lungs are no longer housed in the ribcage after fixation, and the process can take up to 5 weeks (Walsh *et al.*, 2021 ▸). While such imaging is valuable for characterizing lung structures, it still falls short of replicating the natural conditions in which lungs are surrounded by other tissues. To preserve their natural shape and condition, it is therefore best to study lungs *in vivo* or at least *in situ*. Note that *in vivo* studies will introduce potential complications such as motion blur caused by breathing and heartbeat. In contrast, *in situ* experiments may face issues like tissue collapse. The major advantage of excising the entire thorax of the animal is that the lungs would remain in their natural condition, providing a unique opportunity to scan a large-scale lung under conditions that closely resemble the *in vivo* environment.

In this study, we present a method for *in situ* imaging of human-sized animal lungs using the Imaging and Medical Beamline (IMBL) at the Australian Synchrotron. We demonstrate the feasibility of using PBI at photon energies between 50 keV and 80 keV to image entire lungs at a resolution of about 100 µm and with an X-ray dose of 10 mGy, opening up opportunities for future *in vivo* studies in large animal lungs. The use of synchrotron radiation in our experiment provided a high-quality monochromatic X-ray beam with a high degree of spatial coherence, the latter being essential for the in-line phase-contrast imaging method. A direct comparison with standard CT scanners will be performed in a future study.

## 2. Material and methods

### 2.1. Animal preparation

Two whole newborn male calves were obtained from an abattoir. The carcass was prepared by removing the skin and limbs and disarticulating the spine between C1 and C2 and L5, leaving the diaphragm intact. The resultant thorax carcass was inclusive of the ribs, musculature, diaphragm, heart, trachea and lungs, with one weighing 6.40 and the other 6.14 kg. The trachea was intubated (size 8–9 endotracheal tube) and the cuff of the tracheostomy tube was inflated. The excised lungs were then wrapped in a plastic bag to prevent fluid leakage.

### 2.2. Beamline setup

The experiment was carried out in hutch 3B of the IMBL at the Australian Synchrotron. The beamline uses a multipole wiggler, which was set to 3 T, combined with a bent double-crystal Laue monochromator system that produces a parallel monochromatic X-ray beam in the energy range 20–120 keV, with an energy resolution of Δ*E*/*E* ≃ 10^−3^. Imaging was performed at four X-ray energies, 50, 60, 70 and 80 keV, chosen to cover the range of mean X-ray energies used for clinical chest CT. The photon-counting detector EIGER2-CdTe 3M-W (DECTRIS-AG, Switzerland) with a nominal pixel size of 75 µm was used. Each Eiger readout chip contains 256 × 256 pixels, covering an area of approximately 19 mm × 19 mm. Two of these chips are positioned beneath a single slab of CdTe converter material, forming what is referred to as a module.

The Eiger detector used in this experiment consists of three modules arranged horizontally and two modules arranged vertically, creating the full active detection area. However, due to a beam height limitation of 25 mm, only the upper half of the detector was used. There are physical gaps between the modules: horizontally, a 10 pixel-wide gap separates adjacent modules, while vertically the gap is larger – 36 pixels – due to engineering constraints. These gaps are masked out in the image readout and do not contribute to the recorded data. For each photon energy, the detector threshold was set to half of the beam energy. The beamline was set up to use PBI parameters, with a source-to-sample distance of 138.5 m, while the sample-to-detector distance was set to 1, 3, 5 or 7 m. Note that it was not possible to have the detector directly against the sample due to the size of the optical stages. To cover the whole width of the sample, 360° off-center scans with 3600 projections were acquired, with an exposure time of 100 ms per projection, resulting in a total acquisition time of 6 min. Multiple vertical steps were acquired, using a step size of 25 mm, allowing enough overlap (∼12 mm) to subsequently stitch them together using a Python script. This resulted in a field-of-view (FOV) for a single step of 514 × 5800 pixels, and 5000 × 5800 pixels for the whole-stitched volume.

### 2.3. Image pre-processing and reconstruction

Due to the design of photon-counting detectors, there are gaps between the modules caused by the placement of electronics. Moreover, some pixels with a non-ideal response (*e.g.* ‘hot’ pixels) within the detector array can always be found. As a result, it is necessary to perform gap-filling and ‘hot’ pixel correction to address these missing data points. The ‘hot’ pixels were identified using a masking process with the following conditions: (1) the pixel value was above 65000, (2) the number of connected ‘hot’ pixels was less than 10 pixels. The ‘Nearest-neighbor’ interpolator of the *SciPy* Python package was used to correct the image from the ‘hot’ pixels (Virtanen *et al.*, 2020 ▸). For the larger gaps (∼15 pixels) between the detector modules, an in-painting algorithm based on the Navier–Stokes equation (Bertalmio *et al.*, 2001 ▸) was applied in the sinogram space, noting that all the projections were flat-field-corrected prior. Afterwards, the ring removal method from the *Algotom* library (Vo *et al.*, 2018 ▸) was applied to all the sinograms. Details of all the parameters and the functions from the aforementioned libraries can be found in Table 1 ▸. The ‘Paganin’ phase-retrieval algorithm (Paganin *et al.*, 2002 ▸) was applied to all the pre-processed projections (flat-field, gaps and hot pixel corrected), using a δ/β ratio calculated for lung tissue with *xraylib* (Schoonjans *et al.*, 2011 ▸); all the values for the selected energies can be found in Table 2 ▸. The phase-retrieved projections were then reconstructed using a filtered back-projection (FBP) algorithm in the *SYRMEP Tomo Project* (*STP*) software (Brun *et al.*, 2017*b* ▸).

### 2.4. Image quality measurements

To evaluate the quality of the acquired images and optimize parameters for the imaging setup (energy and propagation distance), several metrics were used. The assessment was performed on the reconstructed 2D slices with the same mean absorbed dose for the lung tissue for each energy/distance. In this study, we used the CNR, as described in equation (1): 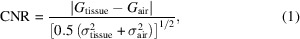
where *G* represents the mean gray value for a region of interest (ROI) of 128 × 128 pixels, for both soft tissue and air, and σ represents the standard deviation of the same ROI. The CNR was measured across four ROIs in the image, and both the average value and its standard deviation were calculated.

**Figure 1:**
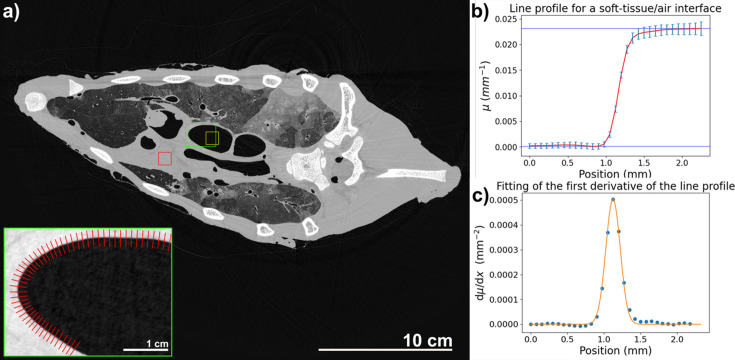
Example of the ROI selection for image quality analysis. (*a*) 2D slice where the red rectangle represents the soft tissue ROI, the yellow rectangle represents the air ROI, and the green rectangle on the soft tissue–air interface is used to perform the line profile extraction. The magnified inset shows the ROI of the interface, and the red lines are the line profiles considered. (*b*) Line profile extracted from the soft tissue–air interface. The blue lines represent the highest and lowest values, soft tissue and air, respectively, while the red profile is the average of all the line profiles considered, and the light blue lines are the error bars. (*c*) The scatter plot depicts the first derivative of the line profile, and the orange line shows the Gaussian fit.

High contrast and low noise are both important aspects in image quality and are therefore often combined into the so-called CNR. Since the TIE-Hom phase-retrieval algorithm inherently acts as a low-pass filter, it reduces noise but also affects spatial resolution. To account for this, we included an additional measurement of edge sharpness, calculated as the full width at half-maximum (FWHM) of the edge profile, for a soft tissue/air interface, as indicated in the detail of Fig. 1 ▸(*a*).

The edge profile, resembling an erf(*x*) function, was differentiated to obtain a Gaussian-like curve. By fitting this first derivative with a Gaussian function, we determined the standard deviation (σ). The spatial resolution (*R*) was then calculated by multiplying σ by a factor of 2.35 [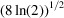] to give the FWHM.

When performing imaging, several performance metrics of the imaging system – such as signal-to-noise ratio (SNR), spatial resolution, and their combination with photon fluence – must be considered. Gureyev *et al.* (2024 ▸) introduced a figure of merit called the intrinsic imaging quality characteristic (*Q*) to capture these factors. However, this metric does not account for the specific sample being imaged. In practice, it is essential to analyze image quality and optimize system performance for a particular class of samples – in this case, lung tissue. One important factor not considered in the intrinsic imaging quality is image contrast, which only becomes relevant in the presence of a sample. To address this, we use the CNR, as defined in equation (1), with the typical goal being to maximize it. Another critical parameter is the radiation dose delivered to the sample during image acquisition. The absorbed dose refers to the energy per unit mass absorbed by the sample and is particularly significant due to the carcinogenic effects of ionizing radiation in medical imaging. To incorporate the sample’s impact on image quality, both CNR and absorbed dose must be included in the overall image quality characteristic, as discussed by Gureyev *et al.* (2025 ▸). To make this figure of merit dimensionless, Gureyev *et al.* (2025 ▸) applied a normalization factor, *R*_ab,air_, calculated in the case of monochromatic radiation as
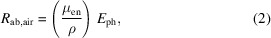
where μ_en_/ρ is the mass energy-absorption coefficient of dry air at room temperature and sea level (Kato, 2014 ▸), and *E*_ph_ = *hc*/λ is the energy of an X-ray photon at the relevant wavelength. However, while the incident kerma (kinetic energy released per unit mass) is typically calculated in 2D, *i.e.* with respect to the 2D incident X-ray fluence, metrics such as CNR and spatial resolution are evaluated in 3D, in the case of 3D imaging such as CT. Therefore, adjustments must be made to account for the 3D nature of imaging modalities such as CT. In CT imaging, the reconstructed sample volume is commonly cylindrical, with radius *R*_c_ and height *H*. The photon fluence is calculated over a flat entrance surface with area Ω = 2*R*_c_*H*. The effective depth *L* of the sample can then be defined as the ratio of the volume of the cylinder to the entrance surface: 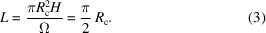
All of these considerations were combined by Gureyev *et al.* (2025 ▸) into a new figure of merit known as the *biomedical image quality characteristic*, *Q*_c_, as defined below:
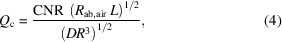
where the spatial resolution *R* is raised to the power of three to reflect that the measurements were performed volumetrically, in agreement with the measurements of the CNR. In contrast, the dose *D* is effectively the sum total of all ‘2D doses’ deposited during the acquisition of each CT projection image (Gureyev *et al.*, 2024 ▸). Fig. 1 ▸(*a*) depicts an example of how the ROIs were selected to calculate the SNR, CNR and spatial resolution using a soft tissue/air interface. The green rectangle shows a magnification of the ROI selected for the calculation of the line profile (red lines), while Figs. 1 ▸(*b*) and 1 ▸(*c*) represent the averaged line profile (red line) used for the calculation and the fitting of the first derivative of the line profile to calculate the spatial resolution, respectively.

### 2.5. Absorbed dose estimation

The air kerma was measured for the incident monochromatic X-ray beam using a Farmer-type ionization chamber (PTW 30013) (Vañó *et al.*, 2017 ▸).

To estimate the absorbed dose in the sample, the dose-to-kerma ratio (DKR) for all tissues in the sample was calculated through Monte Carlo simulations using the CSIRO in-house code [developed by one of the co-authors, Dr Yakov Nesterets (yakov.nesterets@csiro.au)]. These simulations used a voxel­ized 3D model of the sample, with known material composition in each voxel. This model was obtained by manually segmenting a low-noise tomographic reconstruction of the sample into four distinct materials: air, bone, lung and soft tissue. In the original segmented model, the voxel had an isotropic size of about 71 µm. For the Monte Carlo computations, this model was re-binned in all dimensions by a factor of eight (to reduce the computational time), thus producing voxels of side length 0.57 mm.

Trajectories and energy depositions of mono-energetic incident photons were simulated for uniform incident illumination and randomly sampled projection angles in the 360° range. The Monte Carlo computations produced the DKRs for all segmented tissues (ICRU, 1989 ▸), in each voxel of the model. Conversion coefficients from kerma to mean absorbed dose (MAD) were produced for all segmented tissues (see Table 3 ▸). Absorbed doses were obtained by multiplying the incident air kerma by the DKRs. Monte Carlo simulations were carried out for four X-ray energies: 50, 60, 70 and 80 keV.

### 2.6. Software

*SYRMEP Tomo Project 1.6.3* (*STP*) (Brun *et al.*, 2017*b* ▸) was employed for phase retrieval and CT reconstructions. Flat-field correction, in-painting and ring removal were carried out using Python 3.10.14, while spatial resolution calculations were performed with Python 3.12.2. The following packages were utilized alongside Python 3.10.14: *algotom 1.6.0* (Vo *et al.*, 2018 ▸), *numpy 1.24.4*, *opencv 4.10.0.82* and *tqdm 4.66.4*. For spatial resolution analysis, *matplotlib 3.9.0*, *numpy 1.26.0*, *scipy 1.13.0* and *fileswell* (Ahlers *et al.*, 2025 ▸) were applied. *Fiji* was exploited for ROI selection, whereas *VG Studio Max* (2024.2) was employed for data segmentation and 3D rendering.

### 2.7. Hardware

This section contains the hardware details used to process the experimental data. All the raw data were pre-processed using the Australian Synchrotron Computing Infrastructure (ASCI). The reconstruction and the segmentation were run on a Supermicro ws005722 equipped with an Intel Xeon Gold 6326 CPU @ 2.90 GHz to 3.50 GHz and installed RAM of 768 GB (767 GB usable).

## 3. Results

*In situ* imaging of large-scale lungs collected from two calves was successfully performed at the Australian Synchrotron IMBL. In this section we present the results of using the PBI setup at four selected X-ray energies (50, 60, 70, 80 keV) and at four sample-to-detector distances (1, 3, 5, 7 m), to scan one calf lung. For each combination of distance and energy, an image quality analysis using CNR, spatial resolution, bio­medical image quality characteristic (*Q*_c_) was performed. The second calf was scanned over the full height of the lung by performing multiple vertical acquisitions and stitching the reconstructed slices using a Python script.

**Figure 2:**
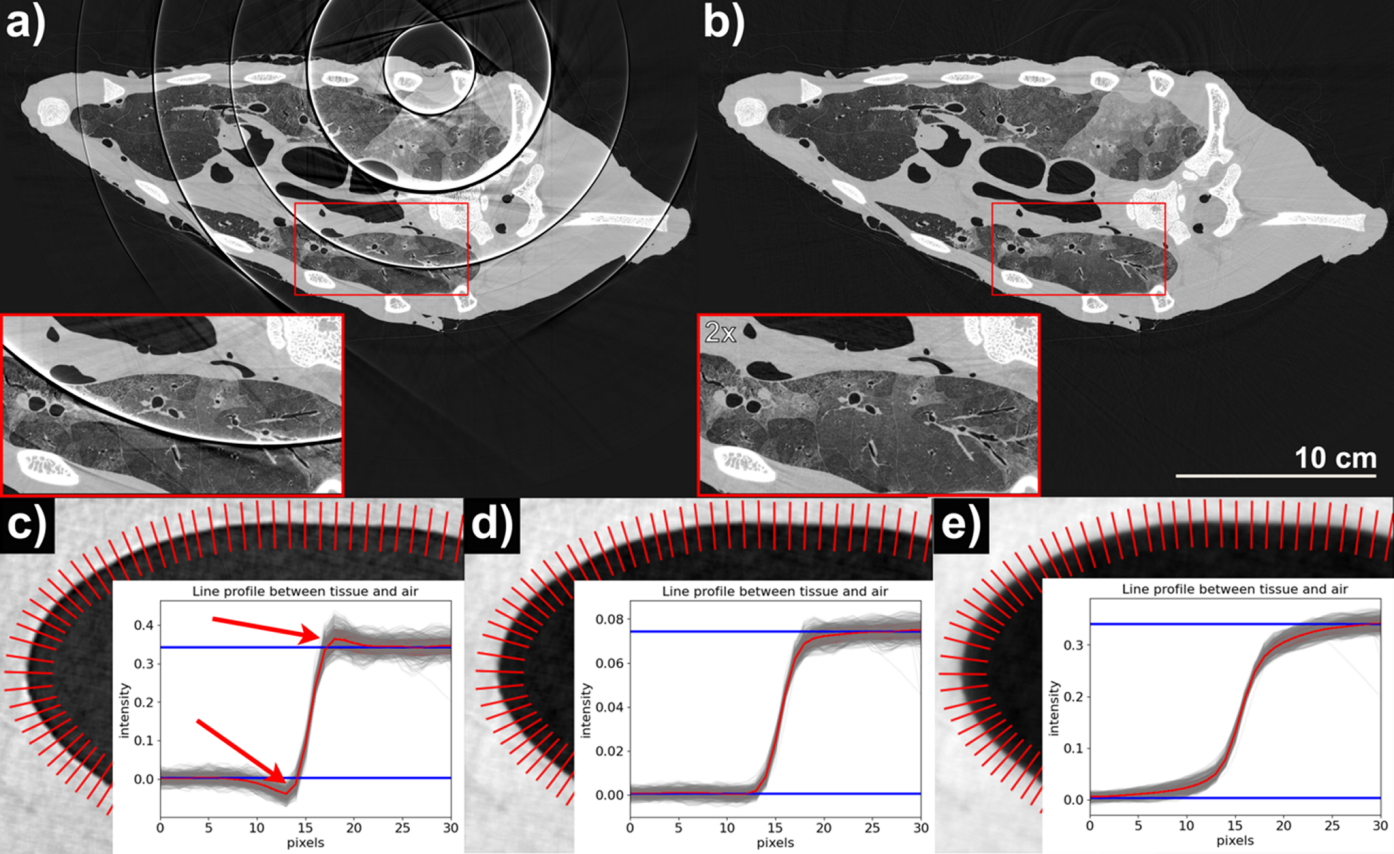
Pre-processing results. (*a*)–(*b*) Reconstructed 2D slice without and with, respectively, the in-painting and the ring removal pre-processing, for 50 keV illumination with 7 m propagation distance. Two magnifications of the same regions are also displayed. (*c*), (*d*), (*e*) Reconstructions with multiple δ/β ratios: half the value calculated with *xraylib* (835), the value calculated (1670) and double the value (3340), respectively. The line profiles show the edge enhancement in (*c*) with the two red arrows indicating the residual phase effects, a correct profile in (*d*), and an over-smoothed profile in (*e*).

Before the reconstruction using the FBP algorithm, the projections underwent pre-processing and subsequent phase retrieval using Paganin’s algorithm (Paganin *et al.*, 2002 ▸). Figs. 2 ▸(*a*) and 2 ▸(*b*) demonstrate the effect of the pre-processing: in particular, Fig. 2 ▸(*a*) shows a CT slice when no gap correction and ring removal were applied, while Fig. 2 ▸(*b*) includes pre-processing as described in Section 2.3.

The combination of in-painting and ring removal suppressed the ring artifacts shown in Fig. 2 ▸(*a*), which were caused by gaps between the modules of the Eiger detector. Figs. 2 ▸(*c*), 2 ▸(*d*) and 2 ▸(*e*) depict an air–soft tissue interface with the line profile of the edge, for multiple values of δ/β. Fig. 2 ▸(*c*) shows the typical edge-enhancement profile due to a lower ratio, while in Fig. 2 ▸(*e*) the ratio is too high, causing an excessive blurring of the image, which is reflected in the overly smoothed edge profile. Fig. 2 ▸(*d*) depicts the value calculated using the material composition and density using *xraylib* (Schoonjans *et al.*, 2011 ▸), as reported in Table 2 ▸. In this example, there are no dents or peaks, red arrows in Fig. 2 ▸(*c*), at the air–tissue interface (edge enhancement) and the edge profile is less smooth, resulting in a sharp, but not oversharpened, image.

**Figure 3:**
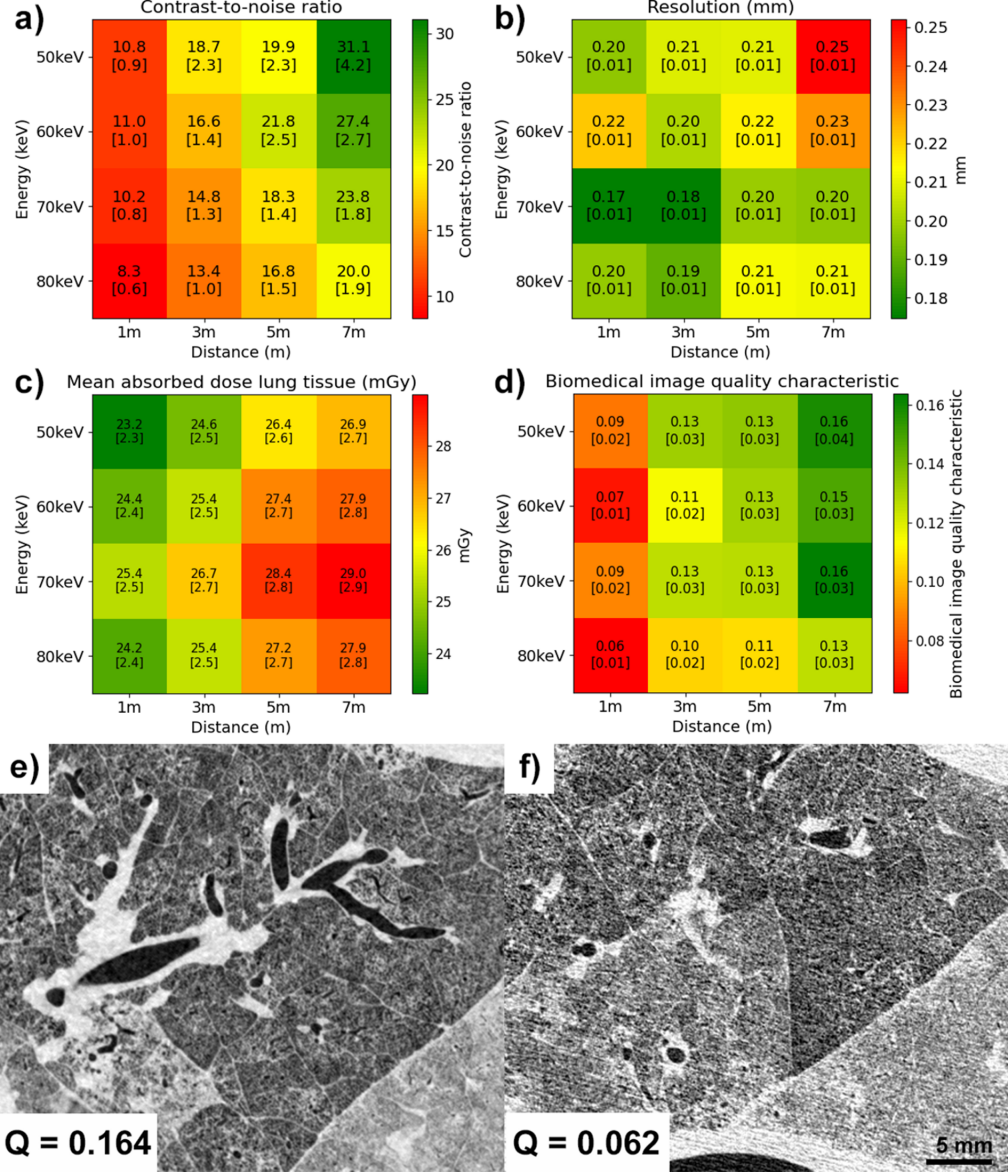
Results of the image quality metrics used to evaluate the imaging setup. (*a*) Contrast-to-noise ratio (CNR). (*b*) Spatial resolution. (*c*) Mean absorbed dose (MAD) in the lung tissue. (*d*) Biomedical image quality characteristic *Q*_c_. (*e*) and (*f*) represent a detail in the slices with the highest (0.164) and the lowest (0.062) values for *Q*_c_, respectively. The uncertainty for each measurement is given in square brackets.

Fig. 3 ▸ depicts the results of the image quality assessment. Images taken with 50 keV X-rays and the largest propagation distance (7 m) have the highest CNR values, while the highest energy (80 keV) with the smallest propagation distance (1 m) has the lowest CNR. The CNR was calculated for four ROIs within the slice. In Fig. 3 ▸(*a*), the reported values represent the average CNR across the four ROIs, along with the corresponding standard deviation. However, due to the rapid beam roll-off [Fig. 6(*a*)], beam inhomogeneities and energy-dependent dose deposition, the spatial location of the slice with a given MAD can vary. Specifically, changes in source-to-detector distance and beam energy affect the beam profile and alignment, and the dose-to-kerma conversion coefficients differ across energies. As a result, the slice corresponding to the same MAD is not always located at the same position within the sample volume. Fig. 3 ▸(*d*) illustrates the biomedical image quality characteristic (*Q*_c_), which combines the metrics of Figs. 3 ▸(*a*), 3 ▸(*b*) and 3 ▸(*c*). Figs. 3 ▸(*e*) and 3 ▸(*f*) depict two magnified regions from images taken at 80 keV, 1 m propagation distance, and 70 keV, 7 m propagation distance, respectively. The two panels display approximately the same regions for the setup with the highest (*Q*_c_) value (0.164), and the lowest (*Q*_c_) value (0.062).

Dosimetry was performed using a Farmer-type ionization chamber (PTW 30013). The dose-to-kerma coefficients, used to convert the incident air kerma to the MAD, were estimated using Monte Carlo simulations on the full volume with partial illumination, where the beam height in the simulation matched that at IMBL. The MAD for each tissue type can be calculated as the product of these coefficients (Table 3 ▸) and the incident air kerma (Table 4 ▸). Table 4 ▸ provides the incident air kerma values for the slice used in the image quality analysis (as evaluated in Fig. 3 ▸) for each energy and distance.

**Figure 4:**
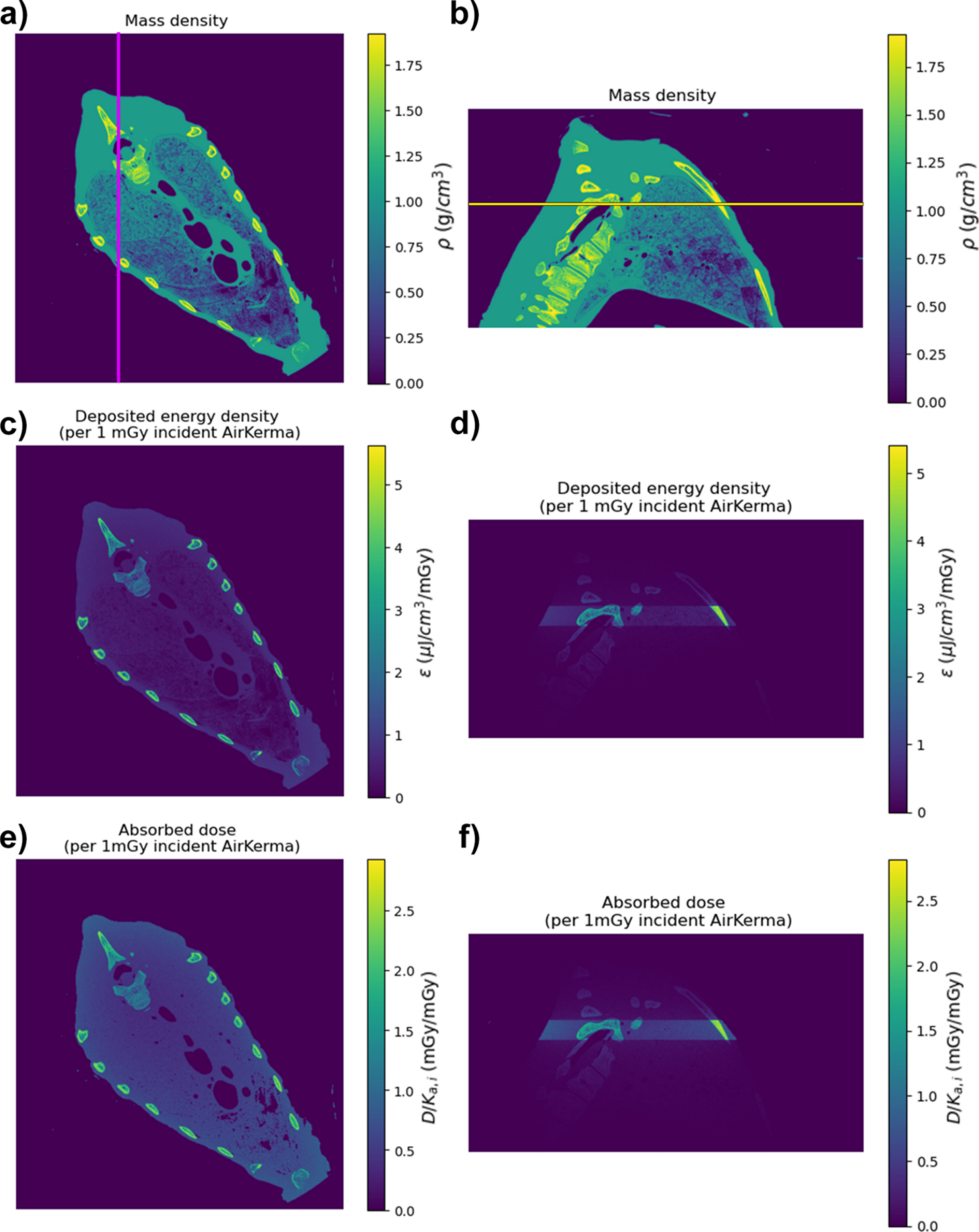
Results of the Monte Carlo simulation for the whole chest volume, for a beam energy of 70 keV. (*a*), (*b*) Mass density distribution, axial and sagittal view, respectively. The purple line in the axial slice indicates the position of the sagittal view. The yellow line in the sagittal view represents the position of the axial view. (*c*), (*d*) Deposited energy per 1 mGy incident air kerma, axial and sagittal view, respectively. (*e*), (*f*) Absorbed dose per 1 mGy incident air kerma, axial and sagittal view, respectively.

Figs. 4 ▸(*a*) and 4 ▸(*b*) present the mass density (ρ) distribution of the whole sample volume, shown with an axial and a sagittal view. The purple and yellow lines in Figs. 4 ▸(*a*) and 4 ▸(*b*) indicate the position of the sagittal and axial view, respectively. Figs. 4 ▸(*c)* and 4 ▸(*d*) display the deposited energy density (ε) per 1 mGy of incident air kerma (*K*_*a*,*i*_). Figs. 4 ▸(*d*) and 4 ▸(*e*) illustrate the absorbed dose (D) for 1 mGy of *K*_*a*,** ***i*_, calculated as the result of Fig. 4 ▸(*c*) divided by Fig. 4 ▸(*a*), and Fig. 4 ▸(*d*) divided by Fig. 4 ▸(*b*), respectively. Table 3 ▸ provides the coefficients to calculate the deposited dose for the whole tissue volume, while Table 5 ▸ yields the coefficients to calculate the deposited energy only in the illuminated region of the whole volume. The fraction of energy deposited within the illuminated region is summarized in Table 6 ▸, which shows that approximately 60–65% of the total deposited dose is confined to the illuminated region, implying that 35–40% of the energy is deposited outside that volume, mainly due to scattering. This demonstrates that the values in Table 5 ▸ underestimate the actual deposited dose in the whole organ by neglecting the scattered energy deposited outside the illuminated area. Table 4 ▸ provides the incident air kerma for the slice used in the image quality analysis (those evaluated in Fig. 3 ▸), for each energy and distance.

**Figure 5:**
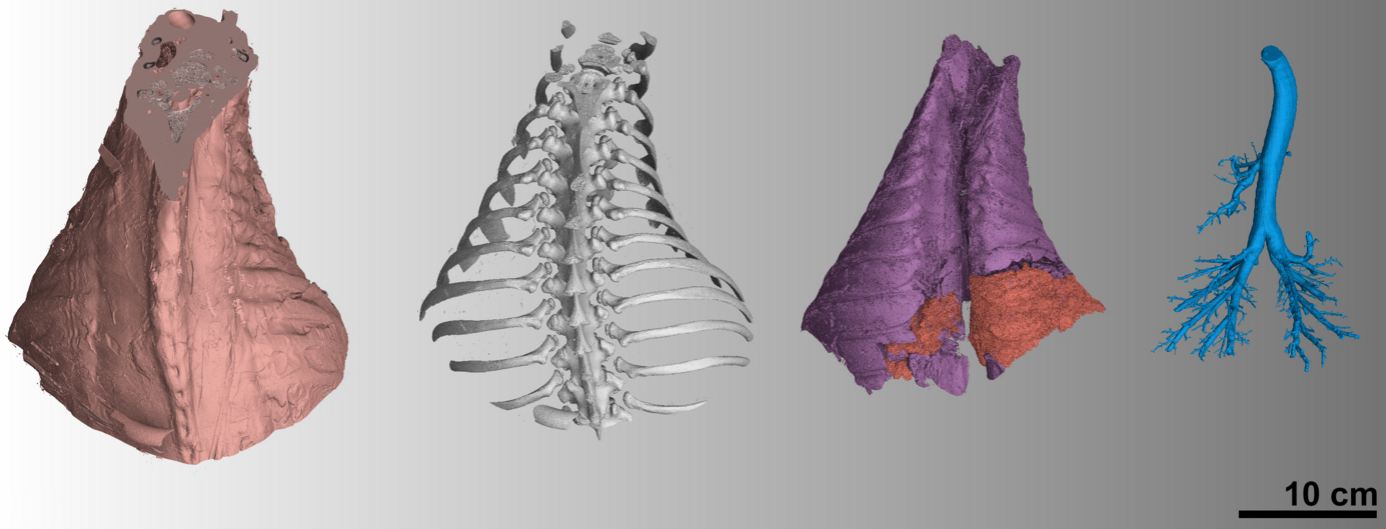
3D segmentation of the reconstructed volume. From left to right: soft tissue, bone, lung tissue (purple fully inflated and orange collapsed), air.

Fig. 5 ▸ depicts the 3D segmentation of the reconstructed volume. It was possible to separate the full volume into four sub-volumes: soft tissue, bone, lung tissue and airways. The lung tissue was also separated into completely inflated tissue (purple) and collapsed tissue (orange). The tissue segmentation shown in Fig. 5 ▸ was used to run the Monte Carlo (MC) calculations using the partial illumination and retrieve the conversion coefficients presented in Table 3 ▸.

**Figure 6:**
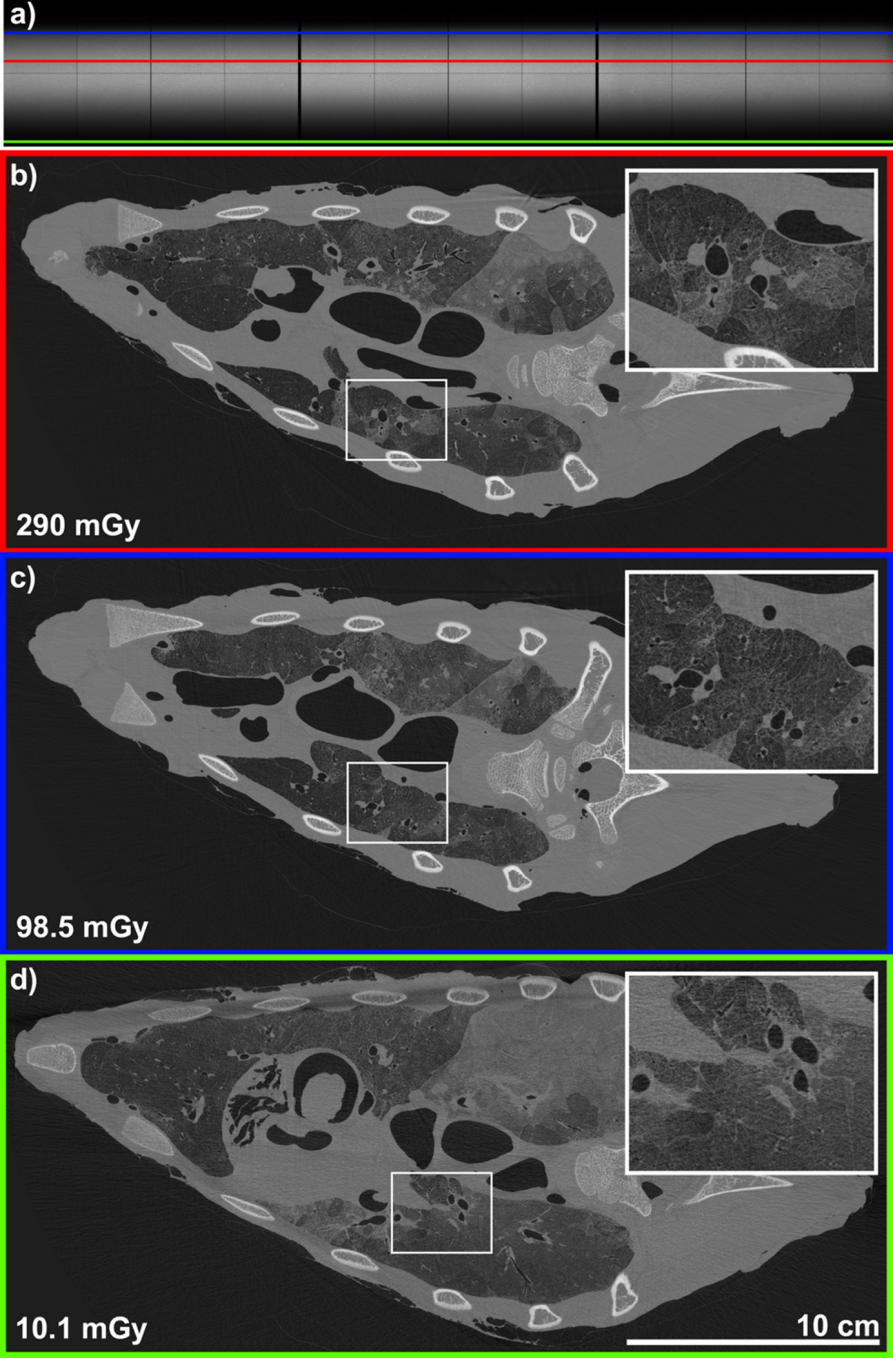
Image quality for different values of the incident air kerma. (*a*) Flat field at 70 keV with 7 m of propagation distance. (*b*) 2D reconstructed slice for an incident air kerma of 290 mGy. (*c*) 2D reconstructed slice for an incident air kerma of 98.5 mGy. (*d*) 2D reconstructed slice for an incident air kerma of 10.1 mGy.

Fig. 6 ▸ represents the image quality of a 2D slice at various positions of the volume, corresponding to varying values of incident air kerma, in the PBI setup with the highest *Q*_c_ (70 keV with 7 m of propagation distance). Because the synchrotron X-ray beam is brightest in the center and reduces in intensity at the top and bottom of the FOV, reduced air kerma is seen at the top and bottom of the reconstructed volume.

## 4. Discussion

In this study, we demonstrated the feasibility of using PBI at the IMBL of the Australian Synchrotron for *in situ* imaging of calf lungs. By employing a photon-counting detector with a nominal pixel size of 75 µm, we achieved a resolution approximately seven times higher than that of high-resolution clinical CT, which typically has a pixel size of 0.5 mm (Tanabe *et al.*, 2018 ▸). However, in some more recent works, researchers have demonstrated that the use of photon-counting detectors could improve the spatial resolution, in clinical scanners, up to 0.17 mm (Thomsen *et al.*, 2022 ▸; Liu *et al.*, 2025 ▸). The energy and propagation distance were optimized for PBI at IMBL using a figure of merit that accounts for image quality (calculated by means of CNR, spatial resolution and dose). Additionally, MC simulations were conducted to determine DKR coefficients, enabling the calculation of the mean deposited dose for each tissue type.

Photon-counting detectors – a recently developed novel type of X-ray detectors – provide high spatial resolution, high quantum efficiency, and the ability to detect single X-ray photons and their respective energy. Photon-counting detectors typically use a hybrid pixel architecture, where each pixel is directly connected to its own readout electronics via bump bonding. This design allows for individual photon counting and energy discrimination at the pixel level. To cover larger imaging areas, multiple detector modules are tiled together. As a consequence, most detectors (such as the Eiger2 used in this study) contain substantial gaps of several pixels between tiles. Therefore, pre-processing of the images prior to reconstruction becomes a crucial step. Several methods have been proposed to solve this issue (Bertalmio *et al.*, 2001 ▸; Criminisi *et al.*, 2004 ▸; Brun *et al.*, 2017*a* ▸; Virtanen *et al.*, 2020 ▸), with interpolation being one of the simplest and easiest to implement. However, interpolation introduces artificial data into the gaps. An alternative approach is to shift the detector during acquisition and then fuse the images to replace the gap region with data of the second acquisition. However, such an approach was not available at the time of the experiment. Here we used an algorithm to in-paint approximately 15 pixel-wide gaps in combination with a ring removal algorithm, which led to non-noticeable artifacts in the reconstructed images.

Phase-contrast X-ray CT is especially suited to lung imaging due to the large differences between the refractive indexes of lung and tissue resulting in strong phase effects (Yagi *et al.*, 1999 ▸). Among the several phase-contrast methods (Wilkins *et al.*, 2014 ▸; Endrizzi, 2018 ▸), PBI seems to be the easiest to implement and, as it does not require any additional optical elements between the sample and the detector, it is a highly dose-efficient method. In addition, phase contrast decreases less rapidly with increasing photon energy than attenuation-based contrast (Gureyev *et al.*, 2009 ▸), which facilitates imaging at higher photon energies and thereby further reducing dose deposition. Despite these benefits, a PBI setup needs careful optimization since image quality is related in a complex way to sample-to-detector distance, photon energy, detector pixel size, the structure causing the phase effect, source spot size and more. We presented an optimization scheme based on rigorous image quality assessment and MC-based dose modeling. The results exploit the unique capabilities of IMBL, reaching high photon energies in combination with a large FOV.

In a previous study performed at the SYRMEP beamline of the Italian synchrotron (Albers *et al.*, 2023 ▸), PBI was used to image human-sized fresh porcine lungs within an anthropomorphic chest phantom using a photon energy of 40 keV in combination with a propagation distance of 10.7 m, which represents the current limits of that beamline. In this study, we aim to explore a wider range of energy and distance combinations. However, due to the design of the IMBL beamline, the maximum propagation distance we could achieve was 7 m. Theoretically, we calculate that the optimal propagation distance for PBI at IMBL, using the Eiger detector, is 12 m (private communication with Timur Gureyev), considering the horizontal source size of 800 µm (Stevenson *et al.*, 2017 ▸). This could be achieved in the future by relocating the sample stage in Hutch 3A. We tested four photon energies, starting at 50 keV and increasing in steps of 10 keV, across various propagation distances ranging from the shortest (1 m) to the longest achievable distance at IMBL (7 m). The former was chosen as the starting point since it provides minimal phase contrast, allowing us to estimate the benefit of phase contrast over attenuation contrast at the synchrotron. In previous studies, several approaches, such as binning (Thurner *et al.*, 2004 ▸), improved sample positioning strategies (Luckow *et al.*, 2011 ▸), and the use of contrast and spatial resolution metrics (Rodgers *et al.*, 2020 ▸), have been employed to optimize imaging parameters for phase-contrast tomography at synchrotron facilities. In the present work, the optimization of the imaging setup was carried out by analyzing image quality using CNR, spatial resolution and a combined metric – CNR, spatial resolution and MAD – introduced by Gureyev *et al.* (2025 ▸) as the biomedical image quality characteristic (*Q*_c_). This approach was chosen specifically because the *Q*_c_ metric incorporates the MAD, a factor not considered in the previously mentioned methods, but essential for optimizing imaging protocols in large animal studies and potential future applications in human imaging. As expected, if the optimization was based solely on CNR, a combination of lower energy (50 keV) and long propagation distances would yield the best configuration. However, when considering spatial resolution and, crucially, dose (since minimizing radiation damage is essential for clinical and sometimes research applications), the biomedical image quality characteristic *Q*_c_ revealed that the optimal setup, for IMBL, utilizes a photon energy of 70 keV and a propagation distance of 7 m.

As a step towards enabling future *in vivo* applications, it is crucial to assess the radiation dose delivered to the animal to minimize radiation damage. For this purpose, one lung was fully scanned using multiple vertical steps for a dosimetry investigation. Dosimetry was performed using a Farmer-type ionization chamber to measure the incident air kerma. MC simulations were conducted on the entire volume with a uniform beam matching the IMBL height to determine DKR coefficients, converting air kerma into deposited dose. Despite partial illumination, energy deposition was observed in adjacent tissues due to scattering. We also calculated the deposited dose only within the partially illuminated region (sub-volume); however, as shown in Table 6 ▸, approximately 35% of the energy deposited in the tissue lies outside the partially illuminated region. This indicates that, for accurate DKR coefficient estimation, the calculation must consider the entire tissue volume rather than only the sub-volume. In this study, no optimization was performed on the exposure time, which was set to 100 ms per projection to ensure sufficient statistics for volume segmentation. This resulted in a total scan time of 6 min, which is challenging for potential *in vivo* applications if breath-holds are required for minimizing motion artifacts. Additionally, the measured incident air kerma for the slice with the highest *Q*_c_ was 290 mGy. Although comparisons with lungs of varying sizes are of limited relevance, Lovric *et al.* (2017 ▸) reported an *in vivo* lung CT experiment in mice with a radiation dose of 334 mGy per projection, resulting in a total dose of 90 Gy for 450 projections per scan, with a reconstructed voxel size of 2.9 µm × 2.9 µm × 2.9 µm (Lovric *et al.*, 2017 ▸). Similarly, Bayat *et al.* (2020 ▸) conducted lung CT studies in small animals, delivering a total radiation dose of 160 mGy for 1000 projections, with a reconstructed voxel size of 320 µm × 320 µm × 600 µm (Bayat *et al.*, 2020 ▸). However, in both studies, the animals were culled, and the long-term effects of radiation exposure were not considered. In terms of PBI of larger animal airways, Donnelley *et al.* (2019 ▸) performed an *in vivo* experiment at IMBL using pigs, but the *in vivo* imaging was limited to a small ROI and involved only 2D projections to track mucociliary transit (MCT) in the trachea (Donnelley *et al.*, 2019 ▸). Full CT imaging was conducted post-mortem, primarily to demonstrate the capability of IMBL to produce high-quality CT images of large animals. They used a CMOS flat-panel sensor with a voxel size of 10.6 µm × 10.6 µm × 10.6 µm, which was less dose-efficient than the photon-counting detector employed in our study. Based on their reported dose rate of 400 mGy s^−1^, and considering 4000 projections with an exposure time of 40 ms per projection, the mean entrance dose per scan was approximately 64 Gy (Donnelley *et al.*, 2019 ▸). Yu *et al.* (2009 ▸) reported that the dose for a sheep undergoing a clinical CT scan is 108 mGy, approximately one-third of the dose in our experiment. However, the goal of our study was to demonstrate the feasibility of *in situ* PBI lung imaging while optimizing photon energy and propagation distance. Using data acquired in the rapid beam roll-off at IMBL, slices with an entrance dose as low as 10 mGy were reconstructed. Our results demonstrate that low-dose imaging can still provide high-quality images, as presented in the *Results* section. Furthermore, with MC simulations, we can now accurately estimate the MAD per tissue based on the measured incident air kerma, thereby supporting the planning of potential future *in vivo* experiments.

Although the X-ray setup is optimized for the current beamline configuration (with an ideal propagation distance of 12 m), sample preparation still requires refinement. Post-mortem effects, such as atelectasis and parenchymal edema, are present in the lung, along with dents between the ribs due to its non-physiological positioning. While post-mortem effects can be eliminated by performing the experiment *in vivo*, the issue of lung deformation must be addressed by scanning the animal in a horizontal position. Before image reconstruction, we were unaware of this issue, but it could potentially be resolved by using an alternative sample stage at IMBL that would allow for horizontal scanning of the calf. However, while this could address the lung positioning issue, it could affect the image quality, since in such a position the transmission will be vastly different depending on the animal’s orientation, due to the asymmetry of the body.

With our optimized imaging approach and dose assessment, we demonstrated the feasibility of low-dose PBI lung imaging, paving the way for future *in vivo* studies. However, post-mortem imaging introduces artifacts due to tissue changes after death and the non-physiological positioning of the chest, which must be considered when interpreting our results. These positioning effects could also affect *in vivo* imaging. To address this in future experiments, the calf or sheep should be positioned horizontally to ensure that the lungs remain in their physiological orientation.

## 5. Conclusion

Based on these promising results, the presented approach could serve as a general method for performing *in situ*, and potentially *in vivo*, lung imaging at the Australian Synchrotron IMBL. This procedure could also be applied in future studies on large animals with known pathologies, such as lung cancer or lung fibrosis, to extend the research conducted on small animals to larger human-sized lungs.

## Figures and Tables

**Table 1:** Parameters and functions used for pre-processing The table reports the methods used, the application type (indicating whether they were applied to projections or sinograms), and the corresponding parameters. For nearest-neighbor interpolation, a ‘–’ symbol denotes the use of default parameters.

Pre-processing method	Application	Parameters
Nearest-neighbor interpolation	Projections	–
In-painting	Sinograms	inpaint(inpaintRadius=10,cv2.INPAINT_NS)
Ring removal	Sinograms	remove_all_stripes(snr=1,la_size=51,sm_size=21)

**Table 2:** Values of the phase shift (δ) and absorption (β) for lung tissue [composition retrieved from Hubbell & Seltzer (2004 ▸)]

Energy (keV)	δ (×10^−8^)	β (×10^−11^)	δ/β (×10^3^)
50	8.77	4.60	1.91
60	6.09	3.39	1.80
70	4.47	2.68	1.67
80	3.43	2.21	1.55

**Table 3:** Conversion coefficients for calculating the mean absorbed dose (MAD) in bone, lung and soft tissue for a measured incident air kerma, in the case of whole-volume partial illumination The table provides the MAD-to-*K*_*a*,*i*_.

	Whole volume		
Energy (keV)	*D*_bone_/*K*_*a*,*i*_	*D*_lung_/*K*_*a*,*i*_	*D*_soft_/*K*_*a*,*i*_
50	0.34	0.085	0.063
60	0.35	0.098	0.072
70	0.33	0.10	0.076
80	0.28	0.10	0.077

**Table 4:** Incident air kerma measured with a Farmer-type ionization chamber (PTW 30013) for 1800 projections

	Incident air kerma (mGy)			
Energy (keV)	1 m	3 m	5 m	7 m
50	258 ± 2.6	273 ± 2.7	293 ± 2.9	299 ± 3.0
60	243 ± 2.4	254 ± 2.5	273 ± 2.7	278 ± 2.8
70	253 ± 2.5	266 ± 2.7	283 ± 2.8	289 ± 2.9
80	242 ± 2.4	254 ± 2.5	272 ± 2.7	279 ± 2.7

**Table 5:** Conversion coefficients for calculating the mean absorbed dose (MAD) in bone, lung and soft tissue for a measured incident air kerma, in the case of sub-volume The table provides the MAD-to-*K*_*a*,*i*_.

	Sub-volume		
Energy (keV)	*D*_bone_/*K*_*a*,*i*_	*D*_lung_/*K*_*a*,*i*_	*D*_soft_/*K*_*a*,*i*_
50	2.4	0.44	0.54
60	2.3	0.50	0.5
70	2.0	0.53	0.62
80	1.7	0.54	0.63

**Table 6:** Fraction of the total deposited energy within the illuminated sub-volume

Energy (keV)	*D*_sub-volume_/*D*_tot_
50	0.65
60	0.63
70	0.60
80	0.60
